# Optimizing Nitrogen Fertilizer Rate and Investigating Mechanism Driving Grain Yield Increase for Rice in the Middle Reaches of the Yangtze River

**DOI:** 10.3390/plants14152326

**Published:** 2025-07-27

**Authors:** Tianxiang Xu, Hailin Zhang, Jie Gong, Ling Wang, Yongsheng Wang, Weiwen Qiu, Muxing Liu, Shenglong Li, Yuanhang Fei, Qi Li, Xin Ni, Jun Yi, Chuanqin Huang

**Affiliations:** 1 Hubei Province Key Laboratory for Geographical Process Analysis and Simulation, Central China Normal University, Wuhan 430079, China; 2 Hefeng County Cultivated Land Quality and Fertilizer Work Station, Enshi 445000, China; 3 Institute of Geographic Sciences and Natural Resources Research, Chinese Academy of Sciences, Beijing 100101, China; 4 The New Zealand Institute for Plant and Food Research Limited, Private Bag 3230, Hamilton 3240, New Zealand; 5 Key Laboratory of Arable Land Conservation (Middle and Lower Reaches of Yangtze River), Ministry of Agriculture, Huazhong Agricultural University, Wuhan 430070, China

**Keywords:** *Oryza sativa* L., grain yield, N fertilizer, N balance, N loss

## Abstract

Investigating the factors influencing rice grain yield (GY) is critical for optimizing nitrogen (N) management and enhancing resource use efficiency in rice cultivation. However, few studies have comprehensively investigated the factors affecting rice GY, considering an entire influence chain encompassing rice N uptake, growth indicators, and GY components. In this study, field experiment with six different N fertilizer rates (0, 60, 120, 180, 225, and 300 kg N ha^−1^, i.e., N0, N60, N120, N180, N225, and N300) was conducted in the Jianghan Plain in the Middle Reaches of the Yangtze River, China, to comprehensively elucidate the factors influencing rice GY from aspects of rice N uptake, growth indicators, and GY components and determine the optimal N fertilizer rate. The results showed that rice GY and N uptake initially increased and then either stabilized or declined with higher N fertilizer rate, while apparent N loss escalated with increased N fertilizer rate. The application of N fertilizer significantly promoted the increase in straw N uptake, which was significantly positively correlated with growth indicators (*p* < 0.05). Among all GY components, panicle number per hill was the most significant positive factor influencing rice GY, and it was significantly positively correlated with all rice growth indicators (*p* < 0.05). In addition, N180 was the optimal N fertilizer rate, ensuring more than 95% of maximum GY and reducing N loss by 74% and 39% compared to N300, respectively. Meanwhile, the average N balance for N180 remained below 60 kg N ha^−1^. In conclusion, optimizing the N fertilizer application in paddy fields can effectively maintain stable rice GY and minimize environmental pollution.

## 1. Introduction

Rice (*Oryza sativa* L.) ranks among the most significant staple crops worldwide, supplying food for more than half of the global population [1,2]. Projection indicated that global rice consumption will reach 590 million tons by 2040 [3]. Nitrogen (N), a critical nutrient for crop growth, is a primary factor that limits rice grain yield (GY) [4,5]. However, there has been limited focus on comprehensively investigating the factors affecting rice GY, considering an entire influence chain encompassing rice N uptake, growth indicators, and GY components. Furthermore, overuse of N fertilizer not only reduces its use efficiency but can also decrease GY and contribute to various environmental problems, including water eutrophication, soil degradation, and increased greenhouse gas emissions [6,7,8,9]. Therefore, optimizing the N fertilizer rate is critical for securing food security and mitigating adverse environmental impacts.

N fertilizer facilitates rice N uptake, thereby enhancing growth and ultimately leading to higher GY [5,10]. Within a specific range of N fertilizer rate, GY tends to rise progressively with increasing N level. However, beyond an optimal rate of N fertilizer, nutrients no longer limit yield [11]. Additional applications of N fertilizer at this point may maintain GY at a relatively stable level or result in a decline [12,13,14]. Key components of GY (i.e., panicle numbers, grain number per panicle, filled grain rate, and 1000-grain weight) directly influence rice GY and are interconnected in intricate ways [13,15]. Likewise, N fertilizer application can promote panicle formation and grain development, while excessive N fertilizer application may lead to over-tillering and stem elongation, which can impede the proper development of panicles and grains [16,17]. Rice growth indicators (such as chlorophyll content, leaf area index (LAI), plant height, and tiller numbers) determine the development of rice panicles and grains [18]. Chlorophyll, a critical photosynthetic pigment, is essential for plant growth and development, as it absorbs and converts light energy [19]. The chlorophyll content, which is closely related to the N content in leaves, operates as an essential indicator of N supply level [20,21]. The LAI, a key measure of plant growth, reflects the capacity of the photosynthetic system [22]. Research has shown that increasing the N fertilizer rate appropriately can increase chlorophyll content and LAI of rice, hence increasing GY [23,24]. In addition, optimal N fertilizer application can increase rice height and tiller numbers, which favors better photosynthesis and GY [16,17]. By contrast, excessive N application may lead to taller and excessively tillered rice and relatively underdeveloped root systems, increasing the risk of lodging and adversely impacting GY [25,26]. However, limited research has analyzed the interrelationships between rice N uptake, rice growth indicators, GY components, and GY, and identified the key factors influencing rice GY.

N fertilizer loss, through processes such as ammonia volatilization, nitrous oxide emissions, N runoff, and N leaching, significantly contributes to low N use efficiency, accounting for 10% to 65% of the applied N fertilizer rate [27,28,29]. These losses not only reduce efficiency but also lead to N pollution, which has broad global impacts, costing between 0.3% and 3% of the world’s gross domestic product [29]. In particular, China experiences the highest N losses in its paddy fields, with an annual average N loss of approximately two million tons, which represents 38% of the N input in these fields [30]. Moreover, N loss increases with a higher rate of N application, and its marginal effect gradually escalates [31]. Consequently, there is an urgent necessity to identify an optimal N fertilizer rate for a specific region that balances rice GY and N loss. This requires a comprehensive evaluation of how the N fertilizer rate affects rice GY and N loss.

The Jianghan Plain, a representative lowland area in the Yangtze River Basin, functions as a vital production base for staple crops, oilseeds, and aquaculture in both Hubei Province and nationwide. Approximately 59.8% of the total cultivated land in this region is dedicated to rice cultivation [32]. Previous research on the impact of N fertilizer rate on rice GY in the Jianghan Plain has focused chiefly on examining the effects from some separate aspects, including rice N uptake, rice growth indicators, and GY components [33,34]. However, studies that simultaneously consider important indicators across all three aspects to comprehensively analyze the impact of N fertilizer rate on rice GY are still relatively scarce. Moreover, limited research has assessed the optimal N fertilizer rate by jointly considering both rice GY and environmental N loss.

In this study, we conducted detailed analyses of the effects of N fertilizer on rice GY, GY components, rice growth indicators, and rice N uptake, drawing on two years of field plot experiments in the Jianghan Plain. Meanwhile, we analyzed the correlations between rice N uptake, rice growth indicators, GY components, and GY. We also thoroughly evaluated the increase in rice GY and apparent N loss at different N fertilizer rates to determine the optimal N fertilizer rate in the study area. The objectives of this study are (1) to clarify the influence of N fertilizer on rice GY, GY components, rice growth indicators, and rice N uptake, (2) to investigate the factors influenceing rice GY, and identify the key determinants contributing to yield variation; and (3) to determine the optimal N fertilizer rates in the study area. This study will provide a theoretical foundation for improving N use efficiency and minimizing N pollution in the Jianghan Plain and surrounding lowland areas of the Yangtze River Basin.

## 2. Materials and Methods

### 2.1. Study Site

**Figure 1:**
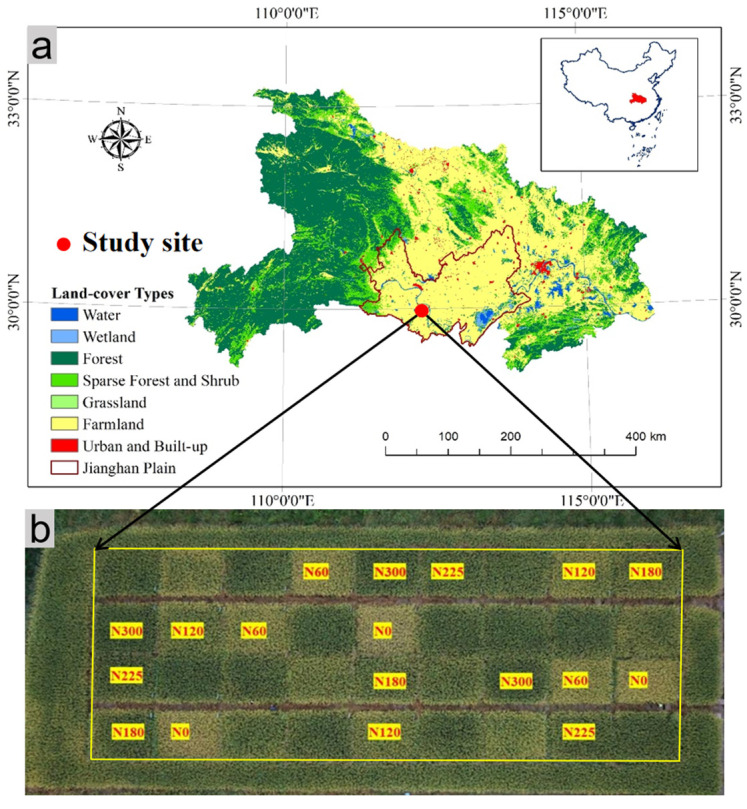
The location of the study area (**a**) and the layout of the experimental plots (**b**).

**Figure 2:**
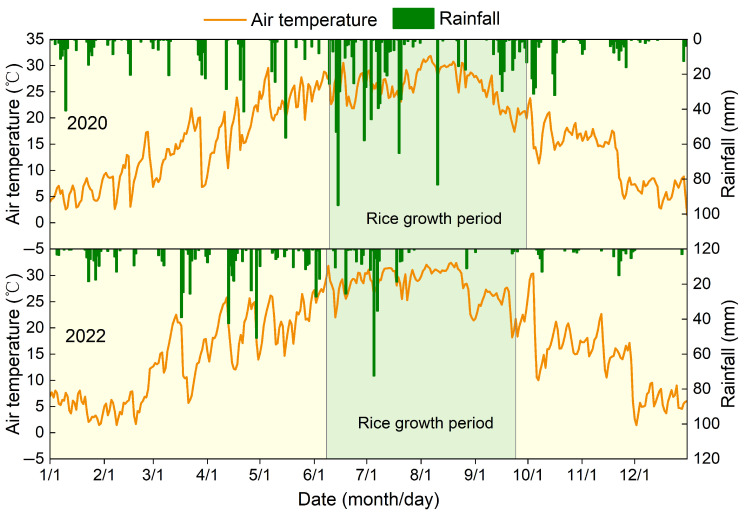
Dynamics of air temperature and rainfall at the study sites, 2020 and 2022.

The Jianghan Plain, located in the central part of Hubei Province, China, has an average elevation of only 27 m. This region is characterized by shallow groundwater levels, typically around 1 m deep, and is dotted with over 300 lakes. The study site is located in the south-central part of the Jianghan Plain (112°34′ E, 29°28′ N; Figure 1a), which falls within the North Subtropical Monsoon Climate Zone. Here, the annual average temperature is 16 °C (Figure 2), and total annual solar radiation ranges from 460 to 480 kJ cm^−2^. The frost-free period lasts approximately 240 to 260 days. It receives an average yearly precipitation of 1000 to 1400 mm, primarily between April and September (Figure 2). The dominant soil is classified as a Typic Endoaquept (USDA Soil Taxonomy), which is characterized by long-term puddling and shallow cultivation and exhibits seasonal saturation. Rice cultivation in the study area typically involves the single-season indica rice that is characteristic of the Jianghan Plain.

### 2.2. Experimental Design and Field Management

In October 2017, an experimental field spanning approximately 1800 m^2^ was arranged in a randomized complete block design in the study area, divided into four columns and nine rows, creating a total of 36 experimental plots (Figure 1b). Each plot had an area of approximately 27 m^2^, and anti-seepage plastic films were used to isolate the plots, preventing lateral movement of water and nutrients through the ridges between plots. In this study, 18 plots were selected for experiments with six N fertilizer rates: 0 kg N ha^−1^ (N0), 60 kg N ha^−1^ (N60), 120 kg N ha^−1^ (N120), 180 kg N ha^−1^ (N180), 225 kg N ha^−1^ (N225), and 300 kg N ha^−1^ (N300). Each treatment was replicated three times, and N300 represented the traditional N fertilizer rate applied by local farmers.

This study was conducted in 2020 and 2022. Rice was transplanted at a density of approximately 19.4 hills m^−2^ with seedling age of 30 days on 9 June, and 8 June across the respective years. Harvesting took place on September 30th and September 24th in 2020 and 2022, respectively. The rice varieties used were a high-yield hybrid variety (Q Liangyou 851) in 2020 and 2022. Urea was used as the N fertilizer in each plot, with a distribution ratio of 35% for the basal application (early to mid-June), 35% for the tillering stage (early July), and 30% for the Jointing–Booting stage (late July to early August). Phosphorus (calcium superphosphate) and potassium (potassium chloride) fertilizers were applied at rates of 40 kg ha^−1^ (P_2_O_5_) and 60 kg ha^−1^ (K_2_O), respectively, only during basal fertilization. Additionally, uniform water management practices were maintained across the plots. Shallow flooding was maintained from transplanting to the late tillering stage, and fields were drained to allow for mid-season drying during the late tillering stage. From the jointing to the grain-filling stage, an alternate wetting and drying irrigation regime was adopted; subsequently, water cessation was implemented during the maturity period.

### 2.3. Sample Collection and Experimental Measurements

Over two years, rice plants from five hills were collected biweekly throughout the growing seasons to measure rice LAI, chlorophyll content, plant height, and tiller numbers. The LAI of rice was measured using a plant canopy analyzer (LAI-2200C, LI-COR Inc., Lincoln, NE, USA). The chlorophyll content in rice leaves was determined using a chlorophyll meter (SPAD-502, Konica Minolta Inc., Tokyo, Japan), which provides a relative measurement of chlorophyll content in soil–plant analysis development (SPAD) units. The heights of ten randomly selected rice plants within each plot were measured using a tape measure. Furthermore, the average tiller number per hill from the sampled rice plants from five hills was recorded. At the rice maturation stage, ten random rice samples were taken from the collected rice plants to measure the grain number per panicle, filled grain rate, and 1000-grain weight after recording panicle number per hill. Subsequently, all the rice from each plot was threshed, and its fresh weight was recorded. Approximately 500 g of grains were selected from each plot for drying to measure the water content and calculate rice GY.

The straw and grains of the rice samples were separately fixed at 105 °C for 30 min, followed by drying to a constant weight at 75 °C before measuring their dry weights. The rice grains and straw samples were digested with H_2_SO_4_–H_2_O_2_, and the released ammonia was distilled and absorbed in boric acid solution using a Kjeldahl nitrogen analyzer (K9840, Hanon Inc., Jinan, Shandong, China), then the absorbed ammonia was titrated with standard sulfuric acid solution to determine the total N content of the samples [35]. In this study, disturbed soil samples were collected from each plot at a depth of 1 m with 10 cm intervals before rice transplantation and after harvest. Additionally, during the rice growth period, one disturbed soil sample was collected every 15 days from each plot at both 0–10 and 10–20 cm soil depths, respectively. Samples of irrigation water and rainfall were also collected. After collection, soil and water samples were immediately frozen for storage. The collected disturbed soil and water samples were analyzed to determine the nitrate nitrogen (NO_3_^−^-N) and ammonium nitrogen (NH_4_^+^-N) content. The NO_3_^−^-N content was calculated based on the difference in absorbance at 220 nm and 275 nm measured using a UV spectrophotometer (TU-1810, Beijing General Analytical Instrument Inc., Beijing, China), and the NH_4_^+^-N content was calculated based on the absorbance at 625 nm measured by the UV spectrophotometer after treatment with the indophenol blue colorimetric method [6].

### 2.4. Data Analysis

#### 2.4.1. Apparent N Balance and N Loss

Due to the difficulties associated with measuring all N input and output pathways, this study opted to calculate the apparent N balance and N loss for different N fertilizer rates, a method that was simpler and more feasible to implement [6,36]. The relevant equations are as follows:

(1) $$\mathrm{Apparent}\;N\;\mathrm{balance}\;(\mathrm{kg}\;N\;{\mathrm{ha}}^{-1})=N\;\mathrm{input}-N\;\mathrm{output}$$

(2) $$\mathrm{crop}\;N\;\mathrm{uptake}\;(\mathrm{kg}\;N\;{\mathrm{ha}}^{-1})=N_{\mathrm{straw}}\times S\;Y+N_{\mathrm{grain}}\times G\;Y$$

(3) $$\begin{array}{l} \mathrm{Apparent}\;N\;\mathrm{loss}\;(\mathrm{kg}\;N\;{\mathrm{ha}}^{-1})=\mathrm{Soil}\;N_{\min}\left(\mathrm{start}\right)+\mathrm{ANM}+N\;\mathrm{input}-\mathrm{Soil}\;N_{\min}\left(\mathrm{end}\right)\\ -\mathrm{Crop}\;N\;\mathrm{uptake} \end{array}$$

(4) $$\mathrm{Yield}-\mathrm{scaled}\;N\;\mathrm{loss}\;(\mathrm{kg}\;{\mathrm{kg}}^{-1})=\frac{\mathrm{Apparent}N\;\mathrm{loss}}{\mathrm{GY}}$$

(5) $$\mathrm{Soil}\;N_{\min}\;(\mathrm{kg}\;N\;{\mathrm{ha}}^{-1})=\frac{\mathrm{Soil}\;\mathrm{thickness}\times \mathrm{Soil}\;\mathrm{bulk}\;\mathrm{density}\times \mathrm{Soil}\;\mathrm{mineral}\;N\;\mathrm{content}}{10}$$

(6) $$\mathrm{ANM}\;(\mathrm{kg}\;N\;{\mathrm{ha}}^{-1})=\mathrm{Soil}\;N_{\min}\left(\mathrm{end}\right)+\mathrm{Crop}\;N\;\mathrm{uptake}-\mathrm{Soil}\;N_{\min}\left(\mathrm{start}\right)-N\mathrm{input}$$

In this formula, N input is the total of N fertilizer plus N derived from irrigation and rainfall, while N output is N uptake by the crop. N_straw_ and N_grain_ represent the N content of straw and grain at the harvest, and SY and GY represent the yields of straw and grain, respectively. Soil N_min_ (start) and Soil N_min_ (end) refer to the amount of soil mineral N (i.e., NO_3_^−^-N and NH_4_^+^-N) accumulation in 0–100 cm soil layers before rice transplantation and after harvest, respectively. ANM, the apparent N mineralization, is calculated using data from N0 and is assumed to be consistent across different N fertilizer rates.

#### 2.4.2. Indices of N Fertilizer Use Efficiency

In this study, N fertilizer recovery efficiency (RE), physiological efficiency (PE), partial factor productivity (PFP), and agronomic efficiency (AE) were used for evaluating N fertilizer use efficiency. The RE reflects the proportion of applied N fertilizer taken up by the crop, while PE indicates the GY produced per unit of N absorbed from N fertilizer. The PFP and AE directly measure the GY obtained per unit of applied N fertilizer without and with blank correction, respectively. The calculations of these indices are as follows [37]:

(7) $$\mathrm{Recovery}\;\mathrm{efficiency}\;(\mathrm{RE},\;\%)=\frac{\left(\mathrm{Crop}\;N\;{\mathrm{uptake}}_{N}-\mathrm{Crop}\;N\;{\mathrm{uptake}}_{N0}\right)}{N\;\mathrm{fertilizer}\;\mathrm{rate}}\times 100\%$$

(8) $$\mathrm{Physiological}\;\mathrm{efficiency}\;(\mathrm{PE},\;\mathrm{kg}\;{\mathrm{kg}}^{-1})=\frac{GY_{N}-GY_{N0}}{\mathrm{Crop}\;N\;{\mathrm{uptake}}_{N}-\mathrm{Crop}\;N\;{\mathrm{uptake}}_{N0}}$$

(9) $$\mathrm{Partial}\;\mathrm{factor}\;\mathrm{productivity}\;(\mathrm{PFP},\;\mathrm{kg}\;{\mathrm{kg}}^{-1})=\frac{GY_{N}}{N\;\mathrm{fertilizer}\;\mathrm{rate}}$$

(10) $$\mathrm{Agronomic}\;\mathrm{efficiency}\;(\mathrm{AE},\;\mathrm{kg}\;{\mathrm{kg}}^{-1})=\frac{GY_{N}-GY_{N0}}{N\;\mathrm{fertilizer}\;\mathrm{rate}}$$

where Crop N uptake_N_ and Crop N uptake_N0_ represent the N uptake by the crop in the N-fertilized treatment and N0, respectively, while GY_N_ and GY_N0_ denote the grain yield for the N-fertilized treatment and N0, respectively.

#### 2.4.3. Data Statistics

The Kruskal–Wallis test (a non-parametric alternative to one-way ANOVA) was used to assess the differences in GY, GY components, growth indicators, soil mineral N content, N balance indicators, and N fertilizer use efficiency indices across different N fertilizer rates, with individual plots considered as the experimental unit. This method was chosen to minimize potential biases resulting from small sample sizes and uncertainties regarding the normality of the data distribution. For post-hoc comparisons, the Mann–Whitney U test was used, and the Bonferroni correction was applied to control the error rate in multiple comparisons. Regression analysis was utilized to model the relationships between N fertilizer rate and both GY and N loss, as well as between GY components and GY, after confirming the assumptions of normality, homoscedasticity, and independence of residuals. Spearman correlation analysis was used to explore the relationships among crop N uptake, rice growth indicators, GY components, and GY. The above analyses were performed using statistical software SPSS 26.0. The experimental data visualizations were obtained using OriginPro 2022.

## 3. Results

### 3.1. GY and Its Components

**Figure 3:**
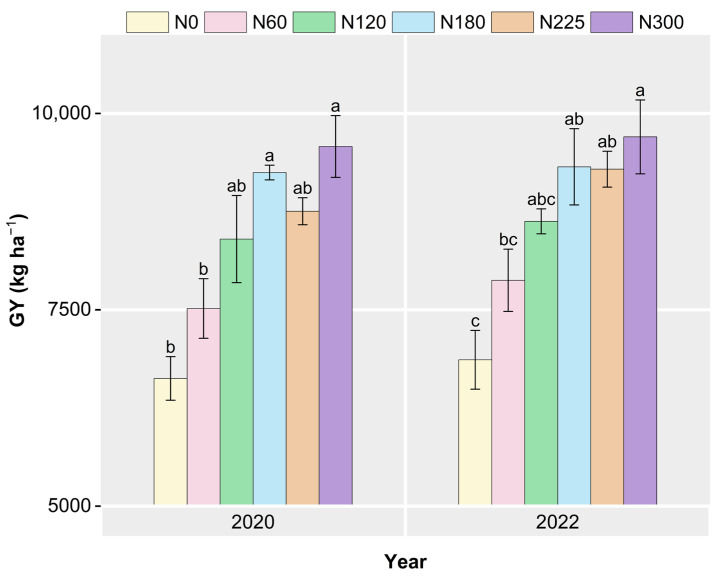
The grain yield (GY) at varying N fertilizer rates in 2020 and 2022. N0, N60, N120, N180, N225, and N300 refer to N fertilizer rates of 0, 60, 120, 180, 225, and 300 kg N ha^−1^, respectively. Identical letters within the same year indicate no significant differences in GY.

As shown in Figure 3, the average GY of N0, N60, N120, N180, N225, and N300 was 6744.8, 7696.0, 8513.06, 9283.2, 9021.9, and 9639.6 kg ha^−1^, respectively. In both 2020 and 2022, the GY was highest with N300, exhibiting a significant increase compared to the yields of N0 and N60 (*p* < 0.05). Meanwhile, no significant differences in GY were observed among N120, N180, N225, and N300 over the two years.

The panicle number per hill was highest at N180 in 2020 and at N120 in 2022. Meanwhile, there were no significant differences in the panicle number per hill among the N120, N180, N225, and N300 over the two years (Table 1). Similarly, the grain number per panicle did not display an increasing trend with the increase in N fertilizer rate, and there were no significant differences in the grain number per panicle of N180, N225, and N300 (Table 1). The trend of the filled grain rate in response to an incremental N fertilizer was an initial ascent followed by a subsequent descent, with the peak of the average filled grain rate (94.0%) observed at the N180. Furthermore, the filled grain rates of N120, N180, and N225 were significantly higher than those of N0 and N300 in 2020, while the filled grain rate of N60, N180, N225, and N300 was significantly higher than that of N0 in 2022 (*p* < 0.05; Table 1). In contrast, no significant differences were observed among the 1000-grain weight of different N fertilizer rates in 2022 (Table 1).

### 3.2. Growth Indicators

**Figure 4:**
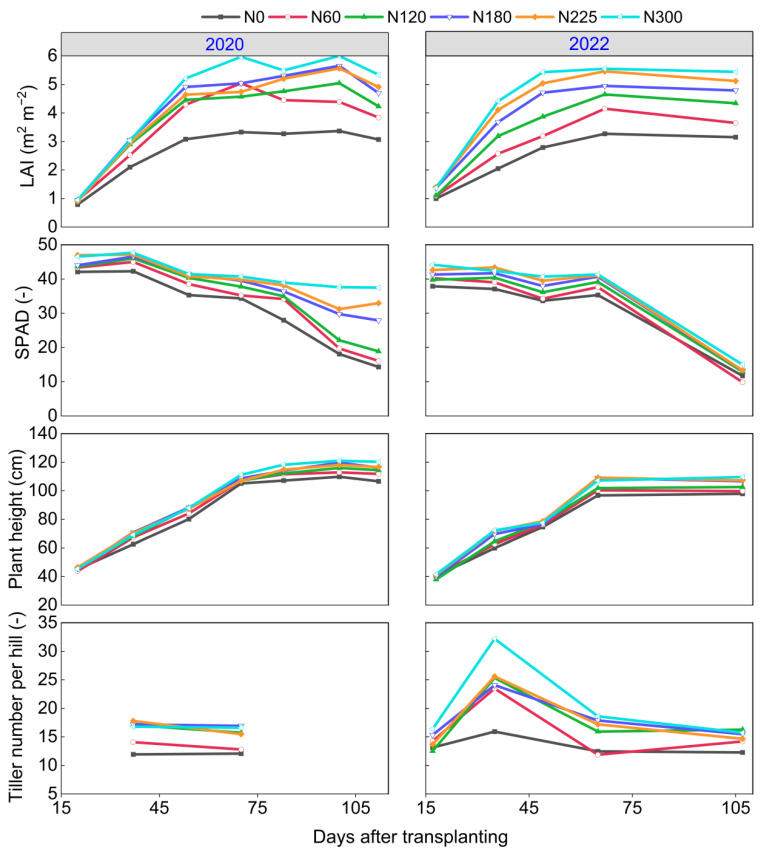
Changes in leaf area index (LAI), soil–plant analysis development (SPAD), plant height, and tiller number per hill over days after transplanting (DAT) at different N fertilizer rates in 2020 and 2022. N0, N60, N120, N180, N225, and N300 refer to N fertilizer rates of 0, 60, 120, 180, 225, and 300 kg N ha^−1^, respectively.

Figure 4 illustrates that the LAI, SPAD, plant height, and tiller number per hill generally increased with higher N fertilizer rate. Both LAI and plant height exhibited distinct increases until approximately 65 days after transplanting (DAT), after which their growth rates either slowed down or began to decline. In contrast, the SPAD gradually decreased during the rice growth period, with a notable decline after 65 DAT. Additionally, a significant increase in tiller number per hill was observed at 35 DAT in 2022, while it exhibited a plateau trend at other times.

### 3.3. Soil Mineral N Content

**Figure 5:**
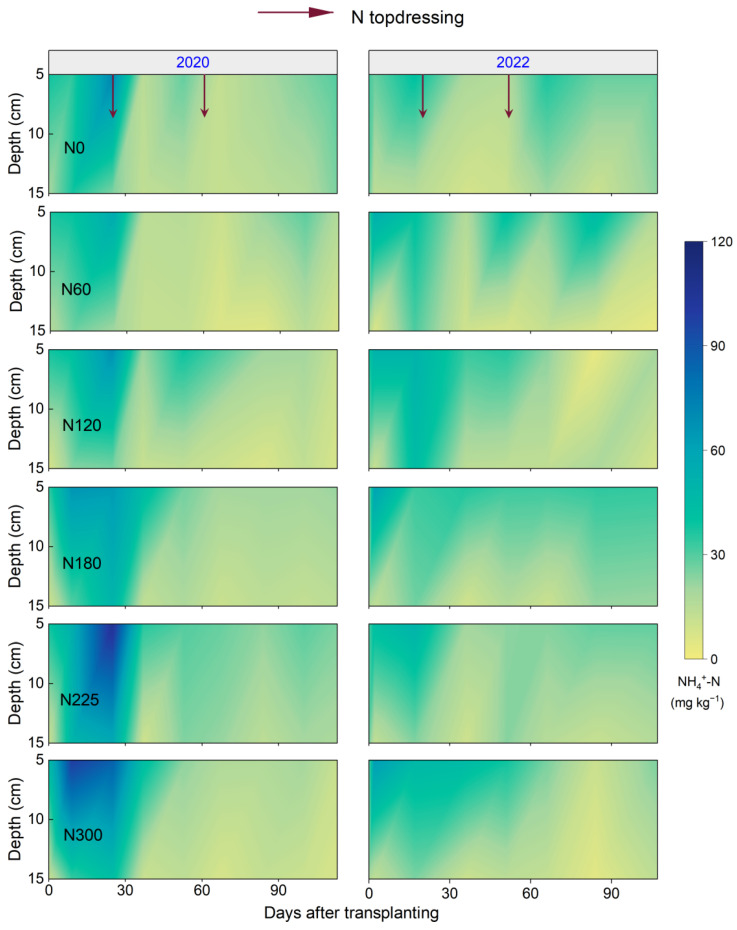
Soil NH_4_^+^-N content at 0–20 cm depth over days after transplanting (DAT) under different N fertilizer rates in 2020 and 2022. N0, N60, N120, N180, N225, and N300 refer to N fertilizer rates of 0, 60, 120, 180, 225, and 300 kg N ha^−1^, respectively.

**Figure 6:**
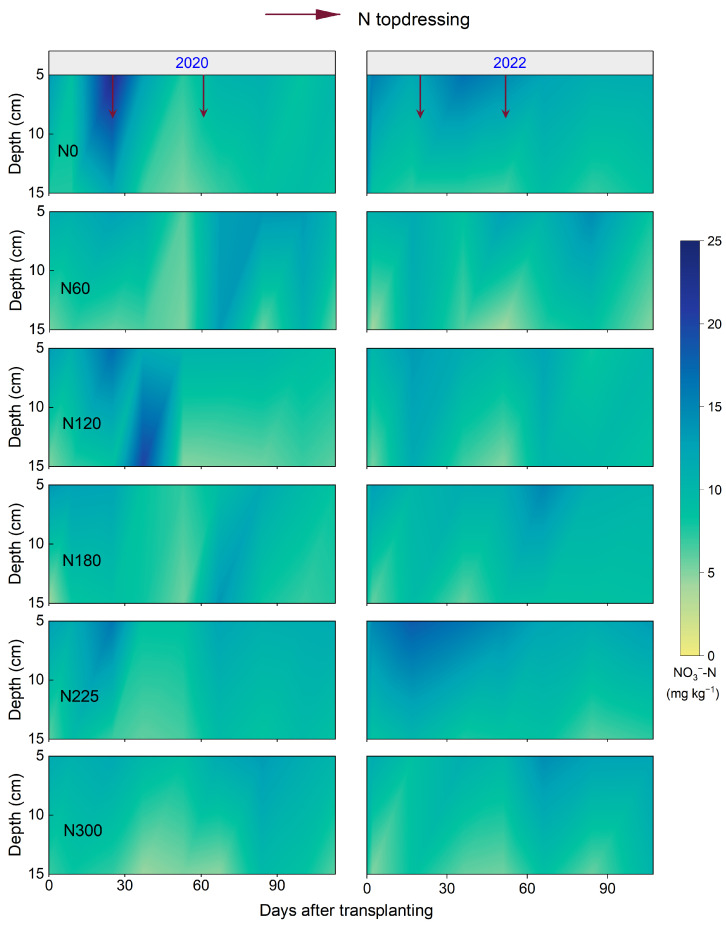
Soil NO_3_^−^-N content at 0–20 cm depth over days after transplanting (DAT) under different N fertilizer rates in 2020 and 2022. N0, N60, N120, N180, N225, and N300 refer to N fertilizer rates of 0, 60, 120, 180, 225, and 300 kg N ha^−1^, respectively.

The soil mineral N content within the 0–20 cm soil layer increased mainly in the days following N fertilizer application (Figure 5 and Figure 6). However, the differences in soil mineral N content among different N fertilizer rates were not significant. Meanwhile, the soil NO_3_^−^-N and NH_4_^+^-N content decreased with increasing depth, with the soil NH_4_^+^-N content (2.6–106.2 mg kg^−1^) being higher than that of NO_3_^−^-N (3.9–23.3 mg kg^−1^). Furthermore, the soil NH_4_^+^-N exhibited more pronounced fluctuations, especially after N topdressing during the rice growth period.

### 3.4. N Balance Indicators

**Figure 7:**
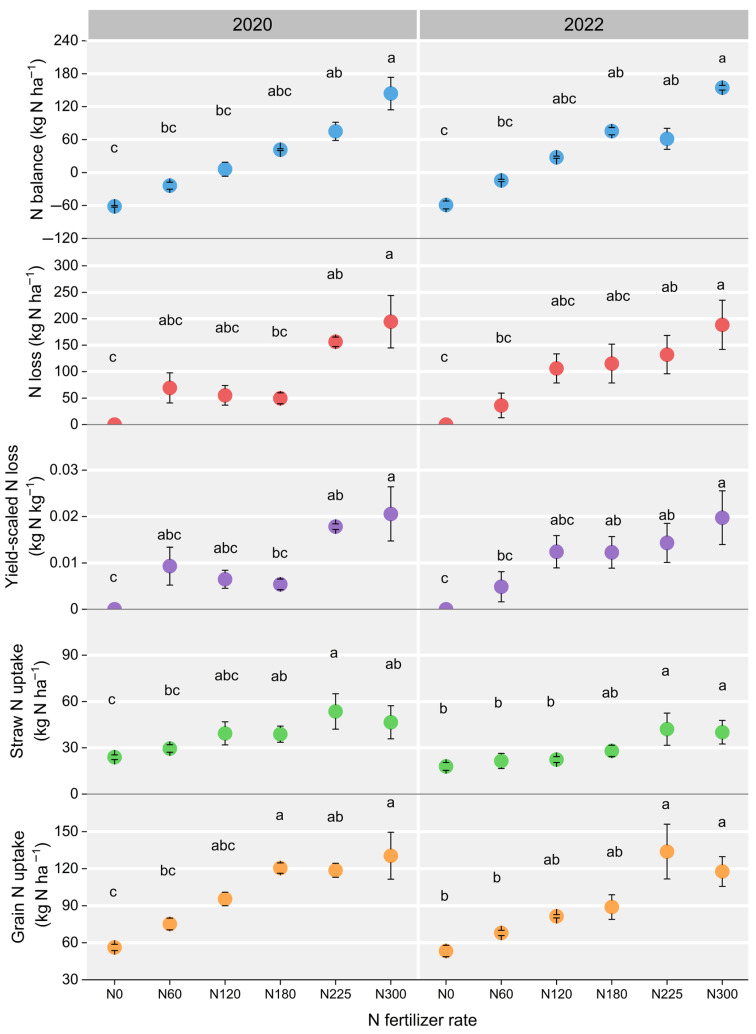
Metrics including apparent N balance, apparent N loss, apparent yield-scaled N loss, straw N uptake, and grain N uptake under different N fertilizer rates in 2020 and 2022. N0, N60, N120, N180, N225, and N300 refer to N fertilizer rates of 0, 60, 120, 180, 225, and 300 kg N ha^−1^, respectively. Identical letters within the same year indicate no significant differences.

The values of apparent N balance, N loss, yield-scaled N loss, straw N uptake, and grain N uptake were significantly influenced by the N fertilizer rate (Figure 7). The N balance value increased following a higher N fertilizer rate, with N300 showing a notable difference compared to N0 and N60 in both 2020 and 2022 (*p* < 0.05). The average apparent N loss of N0, N60, N120, N180, N225, and N300 was 0, 52.7, 80.7, 82.5, 144.3, and 191.5 kg N ha^−1^, respectively, with significant differences (*p* < 0.05) noted between N300 and both N0 and N60 in 2022. Similarly, the apparent yield-scaled N loss exhibited relatively consistent change patterns of apparent N loss across different N fertilizer rates. Moreover, the straw N uptake of N225 was highest in 2020 and 2022, which was significantly higher than that of N0 and N60. Differently, the highest grain N uptake in 2020 and 2022 was achieved at N300 and N225, respectively.

### 3.5. Indices of N Fertilizer Use Efficiency

**Figure 8:**
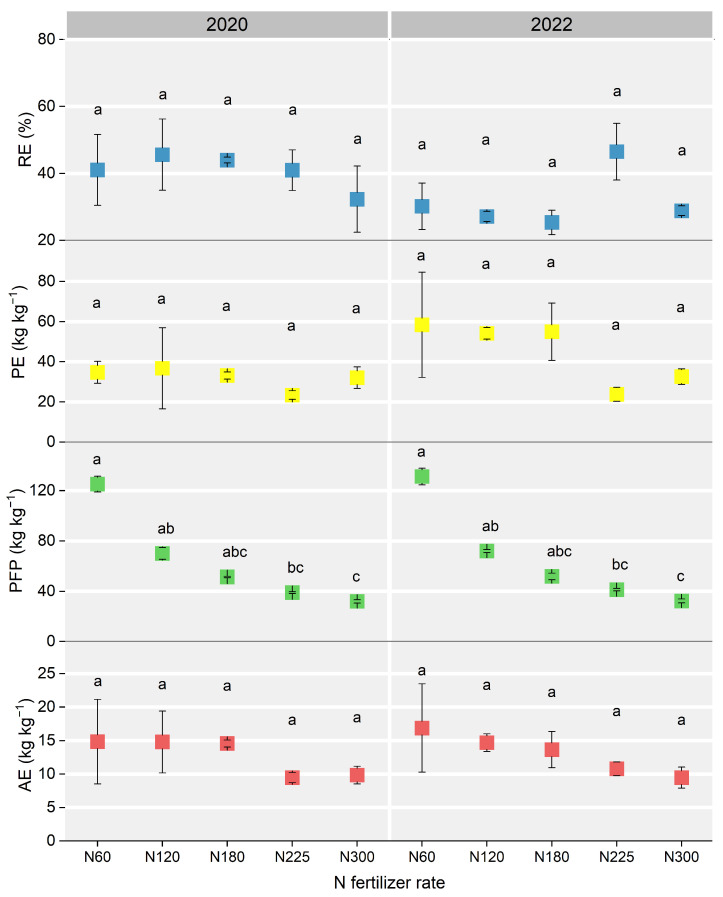
The N fertilizer recovery efficiency (RE), N fertilizer physiological efficiency (PE), N fertilizer partial factor productivity (PFP), and N fertilizer agronomic efficiency (AE) under different N fertilizer rates in 2020 and 2022. N0, N60, N120, N180, N225, and N300 refer to N fertilizer rates of 0, 60, 120, 180, 225, and 300 kg N ha^−1^, respectively. Identical letters within the same year indicate no significant differences.

In this study, the PFP showed a more pronounced decreasing trend with increasing N fertilizer rate compared to RE, PE, and AE (Figure 8). Specifically, the RE values for different N fertilizer rates ranged from 25.3% to 46.5%, with no significant differences among these N fertilizer rates over the two years. Regarding the PE, the highest PE values in 2020 and 2022 were achieved at N120 and N60, respectively, and no significant differences were observed among different N fertilizer rates. The average PFP for N60, N120, N180, N225, and N300 was 128.3, 70.9, 51.6, 40.1, and 32.1 kg kg^−1^, respectively, with N60 and N120 showing significantly higher PFP than N300 over the two years (*p* < 0.05). Furthermore, the highest AE in 2020 and 2022 were achieved at N60, and no significant differences were observed among different N fertilizer rates.

### 3.6. Regression Fitting of N Fertilizer Rate and GY and Apparent N Loss

**Figure 9:**
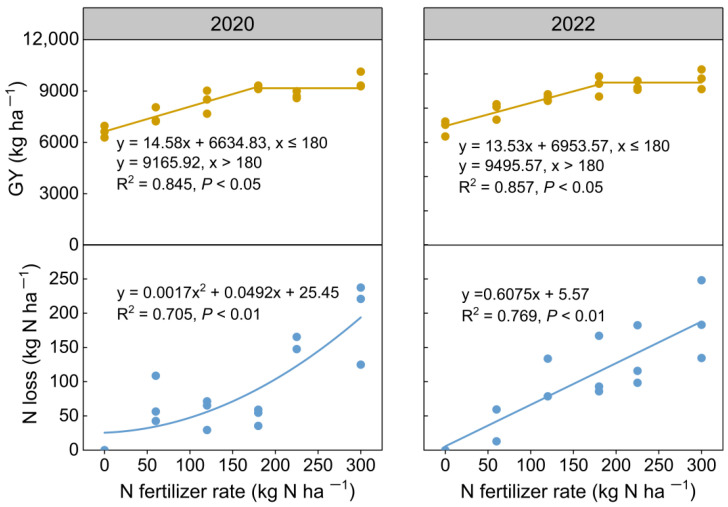
The relationships among N fertilizer rate, grain yield (GY), and apparent N loss in 2020 and 2022.

As illustrated in Figure 9, a significant linear-platform relationship was observed between GY and N fertilizer rate in 2020 and 2022, with GY exhibiting a plateau trend when the N application rate exceeded 180 kg ha^−1^. In contrast, the apparent N loss demonstrated an increasing trend with higher N fertilizer rates, especially in 2020, where the rate of increase in apparent N loss also escalated gradually.

### 3.7. Effect Mechanism of N Fertilizer on Rice GY

**Figure 10:**
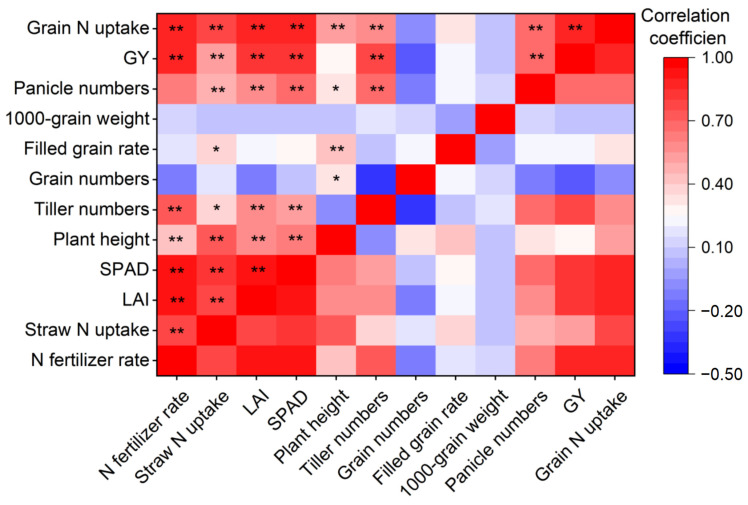
The correlations between rice N uptake, growth indicators, GY (grain yield) components, and GY. LAI: leaf area index, SPAD: soil–plant analysis development. ** indicates significance at *p* < 0.01, and * indicates significance at *p* < 0.05.

In 2020 and 2022, the N fertilizer rate was significantly positively correlated with GY (*p* < 0.01). Panicle numbers, GY, and grain N uptake were significantly positively correlated with one another (*p* < 0.01), while no significant correlations were observed between other GY components and either GY or grain N uptake. The rice growth status during the vegetative growth stage directly influences GY components at the maturity stage. In this study, the panicle numbers showed significant positive correlations with all growth indicators (*p* < 0.05). Furthermore, all growth indicators were significantly correlated with each other (*p* < 0.01), except for plant height and tiller numbers. Straw N uptake was significantly correlated with all growth indicators (*p* < 0.05). Meanwhile, both straw and grain N uptake were significantly positively correlated with N fertilizer rate (*p* < 0.01, Figure 10).

## 4. Discussion

### 4.1. Effects of N Fertilizer on Rice GY and Growth Indicators

Among the essential nutrients for rice cultivation, N is a critical yet frequently deficient nutrient in rice planting systems, and applying N fertilizer is essential for improving rice GY [5,38]. In this study, a significant positive correlation between rice grain N uptake and GY was observed in the two years (*p* < 0.01). However, excessive N application can reduce rice GY and cause severe environmental pollution [12,39,40]. In 2020 and 2022, rice GY exhibited a plateau trend when the N fertilizer rate exceeded 180 kg N ha^−1^, according to the regression analysis results. We also observed that the GY components did not increase significantly with higher N fertilizer rates. Excessive N fertilizer application can inhibit grain filling by reducing the accumulation of cytokinins and auxins, consequently decreasing the rate of filled grains and grain weight [18,39]. In addition, it diverts more assimilates toward vegetative growth, resulting in excessive tillering and stem elongation, which renders rice plants more prone to lodging and leads to poor development of panicles and grains [16,41,42]. The panicle number per hill, grain number per panicle, filled grain rate, and 1000-grain weight are generally considered to be the direct factors affecting rice GY [13,43]. Previous studies have indicated that the panicle number per hill and grain number per panicle significantly impact GY more than other GY components [1,13,44]. In this study, a significant positive correlation was observed only between GY and panicle numbers in 2020 and 2022 (*p* < 0.01), while no significant differences were observed between GY and other GY components. Therefore, panicle number per hill was the key factor influencing rice GY among all GY components in this study.

Rice growth indicators, such as LAI, chlorophyll content, plant height, and tiller numbers, can significantly influence the GY components, ultimately affecting rice GY [17,23,24]. N is a pivotal element for constructing chlorophyll molecules, and increased soil N supply level facilitates chlorophyll synthesis in rice [20,45]. Leaves with high chlorophyll content lead to enhanced photosynthesis, promoting the formation and expansion of leaves, thereby exhibiting a larger LAI. This, in turn, further improves photosynthesis efficiency and promotes tiller initiation and development as well as stem elongation [46,47]. In this study, straw N uptake was significantly positively correlated with growth indicators (*p* < 0.05), and LAI was significantly positively correlated with SPAD (*p* < 0.01). Meanwhile, LAI and SPAD showed significant positive correlations with both tiller numbers and plant height in the two years (*p* < 0.01). Furthermore, tillers were essential for the formation of panicles in rice plants [48], exerting a significant positive effect on panicle numbers, which was also evident in our findings in 2020 and 2022. Therefore, N fertilizer application improved crop N uptake, thus enhancing chlorophyll synthesis and leaf development, thereby boosting photosynthetic efficiency. This improvement stimulated an increase in tiller and panicle numbers, ultimately leading to enhanced GY.

### 4.2. Effects of N Fertilizer Rate on N Balance Indicators

The apparent N balance is a crucial indicator in agricultural N management. A balance value below zero suggests a potential risk of soil depletion, while an excessively positive N balance value indicates an increased risk of N loss [4,14]. In this study, the N balance for N0 and N60 consistently registered values below zero across the two years, indicating that the applied N was insufficient to support rice growth, leading to the depletion of soil N reserves. On the other hand, the N balance for N300 in 2020 and 2022 surpassed 120 kg N ha^−1^, which significantly exceeds the recommended upper limits of 64.9~75.0 kg N ha^−1^ suggested by earlier research [49,50], pointing to a considerable risk of N loss at this rate. Factors such as ammonia volatilization, nitrous oxide emissions, N runoff, and leaching significantly affect the low N fertilizer use efficiency [29,51]. Moreover, the relationship between ammonia volatilization and the N fertilizer rate is linear, whereas other losses exhibit exponential correlations with increasing rates [31,52]. This study also observed an increase in apparent N loss, either quadratic or linear, with rising N fertilizer rate. Furthermore, the patterns of apparent N loss and yield-scaled N loss were similar across different N fertilizer rates, suggesting that the increased N loss due to higher N fertilizer rates outweighed the benefits of increased GY.

Rice primarily absorbs soluble inorganic N (i.e., NO_3_^−^-N and NH_4_^+^-N) from the soil, which constitutes only a small proportion of the total soil N [53]. External N application increases the content of NO_3_^−^-N and NH_4_^+^-N in the soil, thereby promoting the ability of rice to uptake and utilize N [54]. However, the processes of urea hydrolysis, ammonia volatilization, and plant root N absorption occur shortly (a few short days) after applying N fertilizer [55], making it difficult to discern significant differences in soil mineral N content among different N fertilizer rates in this study. Excessive N fertilizer application can also inhibit root growth in both size and length, leaving the root system unable to fully absorb and utilize all available N resources [56]. As the N fertilizer rate increased, grain and straw N uptake showed a tendency to plateau or even decline, signifying that excess N fertilizer was less effectively absorbed by the rice plants, potentially causing greater N loss [57]. An increase in straw N uptake can enhance stem lodging resistance; however, if excessive N remains in the straw and is not effectively translocated to the grains, it may result in inefficient conversion of N into economic GY, thereby reducing N use efficiency [58,59]. The straw N uptake accounted for only 26.1% of the total crop N uptake on average. This phenomenon can be attributed to the cultivation of high-yield hybrid rice in this study, which exhibits significantly higher N demand in grains compared to straw. Consequently, during the later growth stages, a substantial portion of N originally accumulated in straw was translocated to developing grains [17,19].

### 4.3. Optimal N Fertilizer Rate in the Study Area

Determining the optimal N fertilizer rate should account for both economic and ecological perspectives, striving to maintain rice GY while minimizing environmental degradation [52]. In 2020 and 2022, the relationship between GY and N fertilizer rate was best described by a linear-platform curve. The GY increased linearly with the N fertilizer rate up to 180 kg N ha^−1^ and stabilized when the rates exceeded this threshold. Zhang et al. [30] pointed out that the optimal N fertilizer range for rice in China is between 169 and 199 kg N ha^−1^, achieving 95–99% of the maximum rice GY while reducing N loss by 21% to 44%. In 2020 and 2022, the GY of N180 reached 97% and 96% of the potential maximum, respectively, and the apparent N loss of N180 was 74% and 39% lower than at N300. Furthermore, the apparent N loss for N180 and N120 in 2020 and 2022 was notably similar, with differences of less than 10 kg N ha^−1^. N balance is a critical indicator for determining the optimal N fertilizer rate, with a suggested upper limit of 68.8 kg N ha^−1^ for paddy fields in the middle and lower Yangtze River regions [14]. In this study, the average N balance for N180 in 2020 and 2022 was 58.5 kg N ha^−1^, effectively minimizing the risk of soil N depletion and reducing the potential for excessive N loss. Additionally, the PFP of N180 exceeded 50 kg kg^−1^ in both years 2020 and 2022, playing a critical role in mitigating environmental pollution risks associated with N fertilizer use and enhancing N use efficiency in the study area, according to the previous research [49]. Therefore, N180 emerged as the optimal N fertilizer rate for high-yield hybrid rice in this study, sustaining rice GY and simultaneously reducing the environmental pollution from N loss.

In the future, a more precise determination of the N fertilizer rate will be necessary, employing model simulation methods. It is also necessary to determine the optimal N fertilizer rate from an economic perspective, taking into account rice GY income, N fertilizer costs, and the environmental damage costs associated with N loss. Meanwhile, future studies should design field experiments with varied N fertilizer application timings and incorporate measurements of microbial transformation processes (e.g., nitrification and denitrification) to better understand how temporal N availability and microbial activity affect N use efficiency in rice. Considering that water conditions are also a key factor affecting plant growth [60], future research also needs to investigate the combined effects of irrigation and fertilization.

## 5. Conclusions

This study revealed the factors influencing rice GY and determined the optimal N fertilizer rate for single-season rice in the Jianghan Plain. Panicle number per hill was the most significant factor influencing rice GY among all GY components in this study. Increasing the N fertilizer rate significantly promoted straw N uptake, which in turn increased SPAD values and LAI, thus stimulating the rise in tiller and panicle numbers, and ultimately enhancing rice GY. The N fertilizer rate of N180 proved to be the optimal N fertilizer rate, securing over 95% of maximum rice GY while minimizing the risk of N loss and soil N depletion compared to N300. Consequently, optimizing the N fertilizer rate for rice can effectively balance food security with environmental sustainability.

## Figures and Tables

**Table 1:** The grain yield (GY) components at different N fertilizer rates in 2020 and 2022. N0, N60, N120, N180, N225, and N300 refer to N fertilizer rates of 0, 60, 120, 180, 225, and 300 kg N ha^−1^, respectively. If components share the same letter within the same year, it indicates that there are no differences in GY components.

N Fertilizer Level	Panicle Number Per Hill (-)		Grain Number Per Panicle (-)		Filled Grain Rate (%)		1000-Grain Weight (g)
	2020	2022	2020	2022	2020	2022	2022
N0	12.1 ± 2.1 b	12.3 ± 1.7 b	228.5 ± 77.2 a	221.5 ± 58.8 ab	92.4 ± 5.5 b	85.5 ± 6.2 b	19.8 ± 3.4 a
N60	12.8 ± 2.5 b	14.2 ± 3.2 ab	229.1 ± 82.7 a	200.4 ± 60.6 b	93.1 ± 7.4 ab	89.7 ± 4.3 a	18.0 ± 3.1 a
N120	15.7 ± 3.4 a	16.3 ± 3.5 a	222.1 ± 48.0 a	228.3 ± 53.6 a	94.9 ± 5.3 a	89.9 ± 9.9 ab	21.2 ± 2.2 a
N180	16.9 ± 3.3 a	15.5 ± 2.3 a	219.4 ± 48.0 a	201.1 ± 47.1 ab	94.9 ± 5.4 a	93.0 ± 5.7 a	19.4 ± 1.3 a
N225	15.5 ± 3.0 a	14.7 ± 2.1 a	253.4 ± 45.5 a	203.7 ± 39.8 ab	95.0 ± 4.7 a	92.7 ± 3.4 a	19.2 ± 0.3 a
N300	16.6 ± 2.6 a	15.7 ± 3.8 ab	226.0 ± 68.7 a	195.5 ± 60.8 b	90.2 ± 9.8 b	92.1 ± 4.7 a	20.6 ± 4.5 a

## Data Availability

Data are contained within the article.
